# Imipramine and Venlafaxine Differentially Affect Primary Glial Cultures of Prenatally Stressed Rats

**DOI:** 10.3389/fphar.2019.01687

**Published:** 2020-01-31

**Authors:** Ewa Obuchowicz, Anna Bielecka-Wajdman, Michał Zieliński, Grzegorz Machnik, Miłosz Gołyszny, Tomasz Ludyga

**Affiliations:** ^1^Department of Pharmacology, Faculty of Medical Sciences in Katowice, Medical University of Silesia in Katowice, Katowice, Poland; ^2^Department of Internal Medicine and Clinical Pharmacology, Faculty of Medical Sciences in Katowice, Medical University of Silesia in Katowice, Katowice, Poland

**Keywords:** imipramine, venlafaxine, stress, prenatal, primary glial cell cultures, rat

## Abstract

Here, we examine the effects of prenatal administration of two antidepressants—imipramine (IMI) and venlafaxine (VEN)—on morphology and activity of a primary glial culture. Microglia are targeted by antidepressants used for antenatal depression and are important regulators of central nervous system development. In this study, female Wistar rats were assigned to one of four groups: a control group that received water *ad libitum* (1), and groups that received additionally once daily either water (2), IMI (10 mg/kg) (3), or VEN (20 mg/kg) (4) by oral gavage from gestation day 7 to 22. Oral gavage administration induced prenatal stress. Cell cultures were obtained from the brains of 1-day-old pups. Prenatal stress caused a disturbance of sensorimotor function in pups. Prenatal stress also produced alterations in the glial cultures, specifically, an increased percentage of microglia in the mixed glial cultures and an increased percentage of dead cells. Moreover, increased levels of IL1-β, TNF-α, NO, and an increased expression of CX3CR1 mRNA were found in microglia. However, the ratio of Bax/Bcl2 mRNA was reduced. Prenatal stress increased the vulnerability of microglia to lipopolysaccharide (LPS). The mixed glial culture derived from pups exposed to IMI showed greater morphological changes and the highest percentage of microglia. Microglia were characterized by the largest increase in the production of pro-inflammatory cytokines and NO, and the greatest reduction in the expression of CX3CR1 mRNA. Exposure to IMI reduced the effects of LPS on IL-1β production and Bax/Bcl2 mRNA, and exacerbated the effects of LPS on CX3CR1 mRNA expression. Prenatal administration of VEN induced protective effects on microglia, as measured by all studied parameters. Taken together, our data suggest that, by disturbing microglia function, exposure to even mild forms of chronic prenatal stress may predispose individuals to psychiatric or neurodevelopmental disorders. These data also indicate that chronic mild stress sensitizes microglia to immune challenges, which may lead to enhanced neuronal damage in the embryonic brain. The observed detrimental effects of IMI on microglial activity under conditions of prenatal stress may help to explain the teratogenic effects of IMI reported in the literature.

## Introduction

According to the “gliocentric theory,” stress-induced inflammation resulting from microglia activation may trigger a cascade of glial dysfunctions that supports the development of depressive disorders (Czéh and Nagy, 2018). When activated, microglia are reported to produce pro-inflammatory, pro-oxidative, and antineuroplastic processes that are detrimental to function of the central nervous system (CNS) (Garden and Möller, 2006). Preclinical and some clinical findings show that antidepressants suppress microglia overactivation. The suppression of microglia is thought to be responsible, in part, for the mechanism of action of antidepressants since anti-inflammatory effects are critical for the maintenance of neurotransmission, control of synaptic plasticity, and neurogenesis (Kato et al., 2013; Kalkman and Feuerbach, 2016; Zhang et al., 2018).

The present study focuses on two antidepressants: imipramine (IMI) and venlafaxine (VEN) a tricyclic antidepressant, a non-selective serotonin and norepinephrine reuptake inhibitor. IMI is recommended for use as a second-choice drug for the treatment of severe major depression (Stahl, 2017). The potent anti-inflammatory properties of IMI have been demonstrated in several *in vitro* studies using in glial cell cultures, including BV-2 murine microglia, HAPI rat microglia, primary mouse microglia, and rat primary mixed glial cell cultures. IMI was found to substantially reduce the production of pro-inflammatory cytokines (i.e., IL1-β, TNF-α, or IL-6) that were stimulated by lipopolysaccharide (LPS), which is a nonspecific immune activator, or by IFN-γ (Hashioka et al., 2007; Hwang et al., 2008; Song et al., 2012; Obuchowicz et al., 2014). IMI has also been shown to have a weaker suppressing effect on the stimulated secretion of the anti-inflammatory cytokine IL-10 (Obuchowicz et al., 2014) and can decrease NO production (Hashioka et al., 2007; Hwang et al., 2008). To date, only one *in vivo* study has examined the impact of IMI on glial cells. In that study, Kreisel et al. (2014) reported that IMI blocked alterations in the number and morphology of hippocampal microglial cells induced by both short-lasting and chronic unpredictable stress.

VEN is a highly selective serotonin and norepinephrine reuptake inhibitor. VEN is frequently applied as a first-choice drug in the treatment of moderate to severe depression and is also used to treat generalized anxiety disorder and social phobia (Stahl, 2017). To date, only a few *in vitro* studies have examined the impact of VEN on glial cells. In one study using a rat astroglia–microglia co-culture with an increased microglial fraction, VEN was found to induce marked anti-inflammatory effects, including (1) alterations in microglia morphology from the typical activated phenotype to a resting phenotype, (2) enhanced release of the anti-inflammatory cytokine TGF-β, and (3) reduced secretion of pro-inflammatory cytokines IL-6 and IFN-γ (Vollmar et al., 2008). In contrast, one study using a BV-2 cell line exposed to LPS reported only weak dose- and time-dependent anti-inflammatory effects of VEN on TNF-α release and NO production (Tynan et al., 2012). In the same experimental model, Dubovický et al. (2014) found weak anti-inflammatory effects of VEN. The effects of VEN were accompanied by a significant decrease in superoxide production and protective effects on mitochondrial membrane potential and lysosomes (Dubovický et al., 2014).

Results from glial cell cultures are inherently limited in their translational relevance to *in vivo* drug testing. The present study aims to address this limitation. Specifically, this is the first study to investigate phenotypic and genotypic alterations in glial cells derived from rat pups prenatally exposed to IMI or VEN. This is important since antidepressants are used in the treatment of antenatal depression. Depression co-occurs substantially with anxiety and is reported to affect 14–23% of pregnant women (O'Hara and Wisner, 2014). Notably, the number of women taking antidepressants during pregnancy in 2010 was twice as high as in 2002 (Meunier et al., 2013). Given that antidepressants are able to cross both the placental and fetal blood brain barrier, exposure *in utero* may influence the neurological development of the developing fetus. On the other hand, antenatal depression is known to increase the vulnerability to psychopathology in children and in certain cases pharmacotherapy is warranted. Findings from clinical studies concerning the safety of antidepressants and recommendation during pregnancy are equivocal (Muzik and Hamilton, 2016). However, aside from selective serotonin reuptake inhibitors (SSRIs), VEN (Australian Therapeutic Goods Administration, AU TGA, pregnancy category B2) has been recommended for antenatal depression (Bellantuono et al., 2015). IMI (AU TGA pregnancy category C), in contrast, is recommended only when the benefit to the pregnant woman outweighs teratogenic risks (US FDA pregnancy category is not assigned).

In an attempt to address some of the aforementioned issues, here, IMI or VEN was administered by oral gavage, once daily, to pregnant dams from the 7^th^ to the 22^nd^ day of gestation. We investigated the effects of chronic mild prenatal stress induced by the use of the oral administration technique. We also examined the combined impact of chronic mild prenatal stress and administration of IMI and VEN on the dynamics of growth and the morphology of the primary mixed glia cultures. Considering the well-documented importance of microglia for CNS development, and that microglia are a known target of antidepressants, we carried out further studies using separated microglia cultures. We assessed a range of markers including nuclei deformation, cell viability, expression of pro-inflammatory cytokines/apoptotic markers/fractalkine receptor, and nitric oxide (NO) levels in primary microglia cultures under control conditions or exposed to LPS.

## Materials and Methods

### Animals

Wistar rats 2.5 to 3 months old (females, 160–200 g; males, 180–240 g) from the Center for Experimental Medicine of the Medical University of Silesia (Charles River International Genetic Standardization Program) were used in this study. All animals were kept in an animal facility at the Department of Pharmacology under standard conditions (i.e., 12/12 h light/dark cycle, ambient temperature of 22 ± 2^°^C; humidity 55 ± 10%) and had food and water *ad libitum*. After 1 week of acclimatization, female rats were mated with the males overnight (ratio 1♂: 2♀). The presence of sperm in vaginal smears was taken to indicate the probable onset of gestation. Starting at GD 15, the females were housed individually in plastic cages. Experiments were carried out according to the guidelines of the European Directive (2010/63/EU) and were approved by the Local Ethics Committee for the Care and Use of Laboratory Animals (Katowice, Poland).

### Experimental Design

VEN hydrochloride (Alembic Pharmaceuticals Ltd, gift from Adamed, Poland) and IMI hydrochloride (Sigma-Aldrich, USA) were dissolved in distilled water and delivered by oral gavage to sperm-positive females once daily (at 09.00 h) from GD 7 to the last day of pregnancy (0.5 ml/100 g of bodyweight). This approach was chosen to administer the drug prior to the detection of microglia in the brain parenchyma (Alliot et al., 1999). Doses of 20 mg/kg VEN and 10 mg/kg IMI were based on previous studies examining *in utero* exposure of these drugs (e.g., Dubovický et al., 2012; Singh et al., 2015; for review, see Bourke et al., 2014). Female rats were weighed twice a week, and doses were calculated each day according to current animal bodyweight. The dams were assigned randomly to one of four groups. Control rats had free access to bottled water. Rats from the other groups were exposed to a mildly stressful procedure elicited by once daily oral gavage administration of water (prenatal stress), IMI (stress+IMI), or VEN (stress+VEN). Although single oral tube administration of water in male adult rats did not induce a stress response (Brown et al., 2000), it was reported to produce stress in mice (Walker et al., 2012). In our study, prenatal stress in pregnant females caused by single oral gavage drug administration per day was evidenced by alterations in morphology of the primary glial cultures, an increased percentage of dead cells, an increased activation of microglia, and an affected sensorimotor function in pups.

The following experiments were carried out using mixed glial cell cultures and microglia:

*Experiment 1* was performed to estimate the effects of prenatal stress and antidepressants on glia isolated from rat pups. Pups were delivered by five dams from the control group and three dams from each of the other groups (i.e., prenatal stress; stress+IMI and stress+VEN). Mixed glial cell cultures were evaluated by microscopic observations once daily over a 13-day period. Observations were made using the reverse contrast phase fluorescence microscope, Nikon TS-100 F (Japan), and the fluorescence cell analyzer, JuLI (NanoEntec, South Korea). On the last day (day 13), the percentage of microglial cells in the mixed glial cell culture was evaluated using a CD11b/c (microglia rat) MicroBeads magnetic cell sorting analysis (MACS, Miltenyi Biotec, Germany). In the microglia that were separated from the mixed glial cultures on day 13, nuclei deformations and cells with pyknosis were visualized *via* Hoechst staining. Viability of microglial cells that were subjected or not to LPS was determined using (1) lactate dehydrogenase (LDH) release, an (2) MTT conversion assay, and (3) using the cell counter Eve (Life Technologies, USA), equipped with a module to determine cell viability.

*Experiment 2* assessed the effects of prenatal stress and both of the antidepressants (IMI, VEN) on microglial activity, and response to LPS exposure.

In culture supernatants, concentration of the pro-inflammatory cytokines IL-1 β and TNF-α was determined by enzyme-linked immunosorbent assay (ELISA). NO levels were measured using the Griess reaction. Furthermore, expression of mRNA for IL-1 β, TNF-α, and the inducible isoform of NO synthase (iNOS), as well as expression of apoptotic proteins (i.e., proapoptotic Bax and antiapoptotic Bcl2) and CX3CR1 (a fractalkine receptor), was investigated in cellular extracts using reverse transcription and real-time quantitative polymerase chain reaction (RT-qPCR).

*Experiment 3* assessed sensorimotor development of pups. In particular, the surface righting, negative geotaxis, and grip strength tests were performed in pups from the 6^th^ to the 10^th^ postnatal day (for details see behavioral tests).

### Cell Cultures

Primary mixed glial cell cultures enriched with microglia were prepared from the brains of 1-day-old rat pups, and cells were cultured according to previously described methods (see Obuchowicz et al., 2014). In brief, the head of the pup was wiped with 70% ethanol. Then, the pup was euthanized by decapitation with sharp-edged scissors. Brains were then excised aseptically from the skull, separated from the blood vessels and meninges, and mechanically disrupted by trituration in ice-cold Dulbecco's Modified Eagle's Medium (Gibco-BRL, USA). The medium contained 20% heat inactivated fetal bovine serum (FBS) (Gibco-BRL), 100 IU/ml penicillin, 100 μg/ml streptomycin, and 25 μg/ml fungizone (Gibco-BRL). A single-cell suspension was obtained after passing the suspension through needles (0.8 and 0.6 mm) and sterile cell strainers (Becton-Dickinson, USA) with pores sizes of 70 and 10 μm. Next, to obtain a mixed glia culture enriched with microglial cells, the suspension was cultured at high density, namely 1.4 × 10^6^ cells/ml (Saura, 2007). Cultures in 35- or 100-mm poly-_D_-lysine coated Petri dishes (Becton-Dickinson) were incubated at 37°C in an atmosphere of 95% air and 5% CO_2_ for 13 days. The medium was replenished on day 1 after plating, and then every third day with a medium that contained 10% of heat inactivated FBS and the aforementioned antibiotics. Then, 13-day mixed glial cultures grown on 35-mm poly-_D_-lysine coated glass cover slips placed in Petri dishes were fixed using the ethanol–methanol method. Directly before analysis, the cultures were incubated in phosphate buffered saline (PBS) containing 10% horse serum and 1% bovine serum albumin (Sigma-Aldrich, USA) for 40 min on an orbital shaker. Next, cells were incubated overnight at 4°C with fluorescein conjugated *Ricinus communis* agglutinin-1 (1:200) (FL-1081, Vector, USA), which is a lectin that binds to the surface glycoproteins on microglia. After three washes in PBS buffer (pH 7.4) at room temperature in the dark, microscopic analyses were performed (fluorescence microscope Nikon TS-100F). Microglial morphology was evaluated in a minimum of six visual fields for each cover slip. The percentage of microglial cells in the mixed glial cultures was determined using a CD11b/c (microglia rat) MicroBeads magnetic cell sorting analysis (130-105-634 Miltenyi Biotec). In addition, the cultures that were prepared on 35-mm poly-_D_-lysine coated glass cover slips in Petri dishes were subsequently incubated with primary anti-glial fibrillary acidic protein (GFAP) antibody (1:750) (G-3893, Sigma-Aldrich) overnight at 4°C on an orbital shaker. After three washes in PBS (pH 7.4), the cells were incubated for 2 h at 4°C in the dark with fluorescein conjugated secondary antibody (1:500) (AQ503F, Sigma-Aldrich). Then, cover slips were washed three times with PBS and microscopic analyses were performed. The applied high plating density and medium composition did not support rapid growing, proliferation, and maturity of astrocytes. Indeed, the 13-day cultures contained about 30–35% immature astrocytes (i.e., the cultures showed only slight staining for GFAP). No neurons were detected in the cultures, i.e., there were no microtubule associated protein 2 (MAP)-2-positive cells. MAP-2-positive cells were tested for using monoclonal primary antibodies against MAP-2 (ab 5392, Abcam, USA) (1:5,000), followed by a secondary antibody (1:500) (ab 150169, Abcam).

Microglial cell cultures were obtained according to methods described previously (Kowalski et al., 2003). On day 13, the mixed glia cultures plated on poly-_D_-lysine coated 100-mm Petri dish (20 × 10^6^ cells/dish) were shaken in an orbital shaker (200 rpm for 2 h). The suspension of floating cells was filtered through a strainer with pores of 40 μm, and then centrifuged (1,200 rpm, 10 min), suspended in the culture medium, and placed into 96-well plates (5 × 10^4^ cells/well) or 35-mm Petri dishes. Finally, the cells were incubated at 37°C for 30 min. Then, the wells were washed seven times with 200 µl of culture medium to remove non-adhering cells. Microglial cells firmly adsorbed to the bottom (about 92% reacted with *R. communis* agglutinin-1) were incubated overnight before the experiment.

### Hoechst 33342 Staining to Detect Nuclei Deformations, Divisions, or Pyknosis

Hoechst dye 33342 (Sigma-Aldrich) was used to stain the nuclei of microglial cells. Cultures growing on 35-mm dishes were rinsed with PBS and fixed in methanol for 15 min at room temperature, washed again with PBS, and then incubated with Hoechst dye (1 μg/ml) for 20 min at room temperature in the dark. Analysis of cell nuclei was conducted using the Nikon TS-100 F microscope with an excitation wavelength of 350 nm (blue fluorescence). The percent of analyzed cells (i.e., cells with pyknosis, nuclei deformations, or the amount undergoing divisions relative to the total cells) was counted at 40× magnification on at least six randomly selected fields, containing about 100 cells each.

### Assessment of Cell Viability

Viability of cells was evaluated in microglial cultures that were exposed or not exposed to LPS (1 µg/ml) for 24 h. Viability was assessed using (1) an MTT assay, (2) LDH release, and (3) using the cell counter Eve (Life Technologies) equipped with a module for determining cell viability.

In the MTT assay, the ability of cells to convert MTT [3-(4,5-dimethylthiazol-2-yl)-2,5-diphenyltetrazolium bromide] (Sigma-Aldrich) indicates mitochondrial integrity, mitochondrial dehydrogenase activity, and consequently, cell viability (Mosman et al., 1983). To test this, MTT (0.25 mg/ml) dissolved in PBS was added to the culture medium for 3 h at 37°C before the end of the experiment. After washing with PBS, cells were lysed with 100 µl DMSO (Sigma-Aldrich) to allow for dissolution of formazan, a blue reaction product. Absorbance at 590 nm was read on the microplate reader Multiskan RC (Labsystems, Finland).

LDH is rapidly released into the culture medium from necrotic cells. Maximum LDH release is compared with the spontaneous LDH release as determined in the culture medium and cell lysates. Absorbance at 490 nm was read on the microplate reader Multiskan RC (Labsystems).

Data were obtained from three independent experiments (i.e., 3–4 microglia cultures were established from pups of three different dams in each group, for a total of n = 11–12 per group).

### IL-1 β and TNF-α Concentrations in Microglia Cell Culture Supernatants

IL-1 β and TNF-α levels were measured in the supernatant of LPS-stimulated or unstimulated microglia cultures in 96-well plates. LPS (*Escherichia coli* serotype 0111: B4; Sigma-Aldrich) was applied at a concentration of 1 µg/ml. After 6- or 24-h incubation for stimulation of TNF-α or IL-1 β release, respectively (per methods described by Kowalski et al., 2003; Kowalski et al., 2004), the culture medium was harvested and centrifuged (2,000 × g, 5 min). Cytokine quantification was performed using rat ELISA kits (R&D Systems, USA) following the manufacturer's instructions. Absorbance was measured at 450 nm using the plate reader Mulitskan RC (Labsystems). Intra-assay precision, as measured using coefficient of variation for TNF-α, IL-1β was 7.4% and 8.7%, respectively. Sensitivity of determination for both TNF-α and IL-1β was 5 pg/ml. The data were obtained from three independent experiments (i.e., three independent microglia cultures established from pups of three different dams in each group, for a total of n = 9 per group).

### NO Quantification in the Supernatants From Microglia Cultures

Nitrite levels in the supernatant of the microglia cultures from LPS-stimulated vs. unstimulated wells were measured using the Griess reaction. After 24-h incubation with a medium that contained LPS (1 µg/ml), 50 µl of supernatant was collected and mixed with an equal volume of Griess reagent (0.1% N-1-naphthylethylenediamine dihydrochloride/1% sulfanilamide/2% phosphoric acid) (Sigma-Aldrich). The supernatant was placed in a 96-well plate and then incubated for 10 min at room temperature. Absorbance was measured at 450 nm using the plate reader Mulitskan RC (Labsystems). The data were obtained from three independent experiments (i.e., three independent microglia cultures established from pups of three different dams in each group, for a total of n = 9 per group).

### Total RNA Extraction and Reverse Transcription RT-qPCR Analysis in Microglial Cellular Extracts

The expression of mRNA for IL-1 β, TNF-α, iNOS, Bax, Bcl2, and CX3CR1 in cellular extracts was investigated. Microglia cultures were placed on 35-mm Petri dishes (1.4 × 10^6^ cells/dish) and incubated in a medium with or without LPS (1 µg/ml). Expression of TNF-α mRNA was estimated in cultures exposed to LPS for 4 h. The expression of IL-1 β, iNOS, Bax, Bcl2, and CX3CR1 mRNA was estimated in cultures treated with LPS for 12 h. After incubation, the medium was removed, cells were washed with cold PBS, and then cold TRI Reagent was added for 10 min. Next, the cells were scraped, vortexed, collected in sterile Eppendorf tubes, and left on ice for 20 min. All ribonucleic acids were extracted from cells using TRI Reagent according to the 1-step extraction Chomczynski method described in protocols by Ribaudo et al. (1999). RNA precipitates were finally dissolved in 100 µl of nuclease-free water and quantified spectrophotometrically at 260 and 280 nm. Next, 1 µg of total RNA was reverse-transcribed into complementary DNA using the High-Capacity cDNA Reverse Transcription Kit (Life Technologies Polska, Poland) in a total volume of 20 µl per reaction. The thermal profile consisted of exposure to 25°C for 10 min, 37°C for 120 min, and 85°C for 5 min. Finally, the reverse transcription reaction mixture was diluted at a 1:4 ratio with RNAse-free water. Samples were stored at -20°C prior to performing quantitative gene analysis.

Quantitative analysis of gene expression was carried out by a RT-qPCR assay based on SYBR Green I chemistry (Life Technologies Polska, Poland). RT-PCR was performed using PowerUp™ SYBR™ Green Master Mix (ThermoFisher Scientific, Poland). The reaction mixture contained 10 µl of qPCR Master Mix in a total volume of 20 µl, and 200 nmol/l of each forward and reverse primer. The selected genes (i.e., IL-1β, TNF-α, iNOS, CX3CR1, Bax, and Bcl2) were analyzed against a reference gene (i.e., GAPDH). Primer sequences were derived from a RT-PCR primer database (http://www.rtprimerdb.org/). The relative quantification experiment type was chosen for the interpretation of qPCR analysis. Quantitative PCR was performed on a Roche Light-Cycler 480 RT-PCR system (Roche Diagnostics Polska, Poland). The reaction’s thermal profile was as follows: 95°C for 3 min, 40 cycles of 95°C for 35 s, 57°C for 15 s, and 72°C for 1 min. Thereafter, a melting-curve analysis was conducted to confirm the specificity of each reaction. The amount of measured transcript in a sample, normalized to an endogenous reference and relative to a calibrator, was calculated using the 2^-ΔΔCt^ method.

The data were obtained from three independent experiments (i.e., three independent microglia cultures established from pups of three different dams in each group, for a total of n = 9 per group).

### Behavioral Tests

In both male and female pups, Fox's battery of tests was carried out according to Feather-Schussler and Ferguson (2016). The battery of tests included (1) surface righting, (2) negative geotaxis, and (3) grip strength tests.

In the surface righting test, the time taken for a pup to right itself from a supine position was measured. This reflex was evaluated on postnatal day 6. The negative geotaxis reflex, which assesses motor coordination in young rodents, was performed on day 8. The time taken for a pup placed facing down a slope (45^0^ incline) to reorientate itself after release was measured. In the grip strength test, the latency for the pup to fall from the fiberglass screen wire after inversion to 180° was measured on day 10.

### Statistical Analysis

Statistical analyses were performed using GraphPad Prism 7.04 software system (GraphPad Software Inc., San Diego, CA). Normality of the distribution of variables was assessed using the Shapiro–Wilk test. For comparisons that included more than two groups, significant group differences were evaluated using a one-way ANOVA followed by Bonferroni *post hoc* tests. Data that were not normally distributed were analyzed using the Kruskal–Wallis test, followed by Dunn's *post hoc* test. Differences between two groups (e.g., control vs. control+LPS) were assessed using Student's *t*-test or the Mann–Whitney *U* test where appropriate. Data are expressed as mean ± standard error of the mean (SEM). Group differences that reached *p* < 0.05 were considered to be statistically significant.

## Results

### Effects of Mild Prenatal Stress and Antidepressant Treatment Combined With Stress on Pregnancy Outcome and Pup Sensorimotor Functioning

There were no effects of prenatal stress or IMI/VEN treatment combined with stress on gestation period, and all pups delivered on day 23. Dam weight gain from GD 7 to GD 22 was similar across all groups. Although there was a similar number of pups in the litters across the groups, pup bodyweight on postnatal day 1 differed (*F*_(3,180)_ = 32.57, *p* < 0.0001, one-way ANOVA). In particular, the bodyweight of IMI-exposed pups was lower than in other groups (*p* < 0.0001). We also estimated some parameters of neuromotor and reflex development in the offspring on days 6–10. Time of the surface righting reflex differed across groups (*p* < 0.0001, Kruskal–Wallis test). In particular, the surface righting reflex was delayed in pups of dams that received water by oral gavage (i.e., the prenatal stress group, *p* < 0.05) as compared to the control group. Further, treatment with either IMI or VEN normalized the reflex as compared to the control pups. VEN was associated with a stronger normalization effect as compared to IMI. We also found that prenatal exposure to IMI markedly reduced grip strength vs. control (*p* < 0.05, Kruskal–Wallis test) (**Table 1**, **Supplementary Table 1**).

**Table 1:** Pregnancy outcomes and sensorimotor function of pups.

	CONTROL	PRENATAL STRESS	STRESS^+^ IMI	STRESS+VEN
Pregnant dams (n)	7	5	5	5
Dam's weight gain (g)	60.5 ± 3.5	58.5 ± 8.5	67.5 ± 2.5	60.4 ± 2.5
Pup's body weight (g)	6.3 ± 0.1	6.6 ± 0.1	5.4 ± 0,1^ 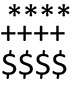 ^	6.4 ± 0.1
Number of pups per litter	9 ± 1	8 ± 1.4	8.6 ± 0.9	9.2 ± 0.9
Surface righting (s) *6 PND*	1.7 ± 0.3	3.4 ± 0.5^*^	1.7 ± 0.2^+^	1.3 ± 0.1^++++^
Negative geotaxis (% of passed) *7 PND*	79.0 ± 11	53.0 ± 13	67.0 ± 16	71.0 ± 12
Grip strength (% of passed) *10 PND*	93 ± 0.7	80.0 ± 10	44.0 ± 17^*^	79.0 ± 11

During pregnancy dams were drinking water (control group) or additionally received water (prenatally stressed group) or IMI (10 mg/kg) (stress+IMI group) or VEN (20 mg/kg) (stress+VEN group) by oral gavage once daily from GD 7 to GD 22. Values are presented as the mean ± SEM. Data of negative geotaxis and grip strength tests are expressed as percentage of pups that had positive result during the testing time. Results concerning pup's body weight were analyzed by one-way ANOVA followed by Bonferroni post hoc test: **** p< 0.0001 vs. control group, ^++++^ p< 0.0001 vs. prenatal stress group, ^$$$$^ p < 0.0001 vs. VEN group. Behavioral data were analyzed by Kruskal–Wallis test followed by Dunn's test: * p < 0.05 vs. control group; ^+^ p < 0.05, ^++++^ p < 0.0001 vs. prenatal stress group.

### Glial Cultures Prepared From Offspring of Pregnant Dams Given Water *ad libitum*

**Figure 1:**
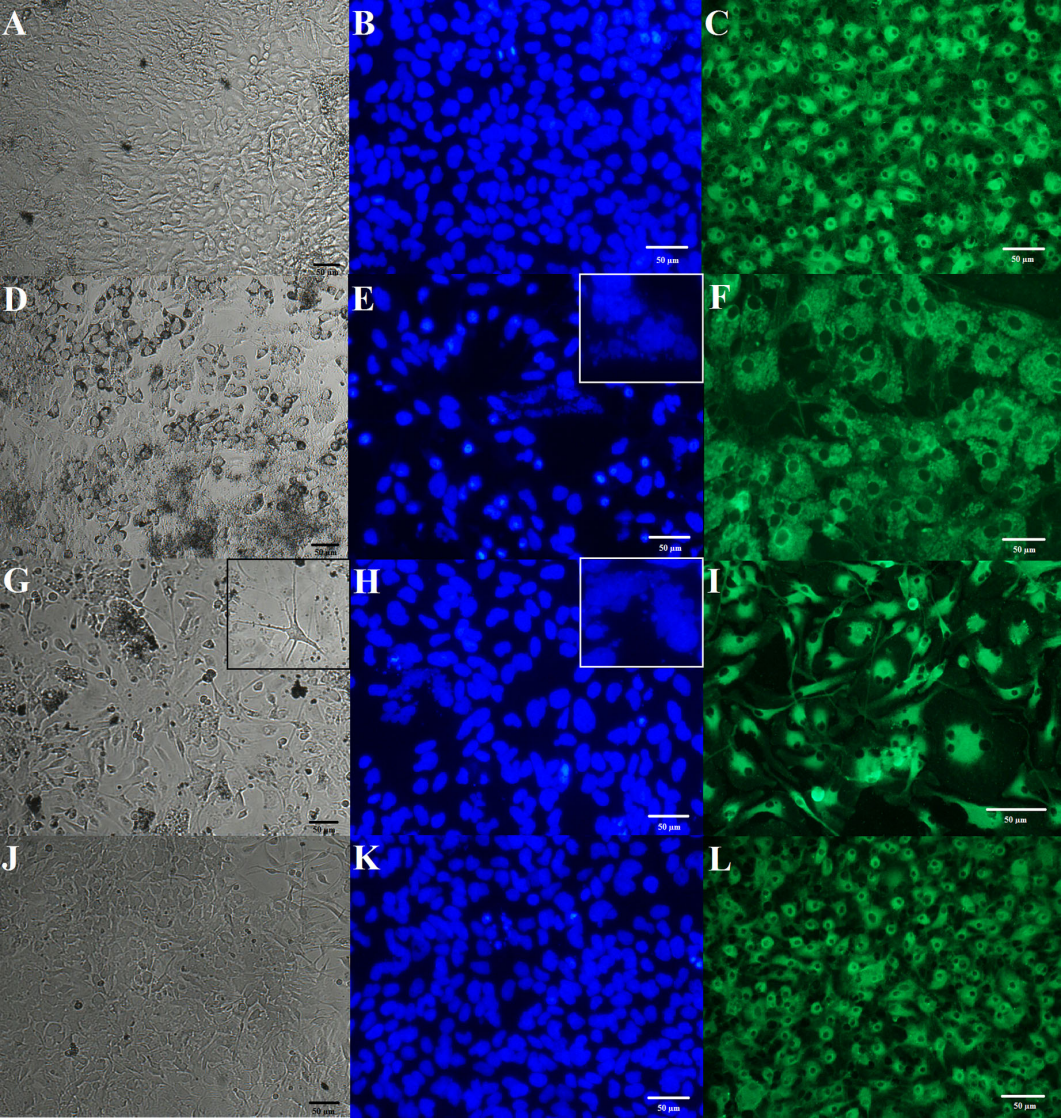
Effects of prenatal stress and administration of imipramine (IMI) or venlafaxine (VEN) during pregnancy on morphology of mixed glial and a microglia culture. IMI and VEN were administered *via* oral gavage. Representative images of mixed glial culture on day 8 in the bright field (using cell analyzer JuLI, magnification 20×) are shown for **(A)** control group, **(D)** prenatal stress group, **(G)** stress+IMI group, and **(J)** stress+VEN group. Representative phase contrast images were taken on microglia separated from the mixed glial culture on day 13. Cellular nuclei were stained using Hoechst 33342 (blue), using a fluorescent microscope (Nikon TS-100F, magnification 40×): **(B)** control group, **(E)** prenatal stress group, **(H)** stress+IMI group, and **(K)** stress+VEN group. Microglial cells were stained with fluorescein conjugated agglutinin-1 (*R. communis*), which is a marker of surface glycoproteins on microglia (green), using a fluorescent microscope (Nikon TS-100F, magnification 40×): **(C)** control group, **(F)** prenatal stress group, **(I)** stress+IMI group, and **(L)** stress+VEN group. For interpretation of the references to color in this figure legend, please see the web version of this article.

In the mixed glia culture, cells became adherent beginning at the fourth or fifth day of culture. Morphology of cells in the culture was typical for a mixed glial cell culture and included ramified forms of cells with pseudopodia and some round-shaped cells. Cells exhibited a tendency to contact each other, and they grew as a monolayer on the bottom of a dish. The cells did not detach from the dish bottom and they did not form aggregations. On the ninth day, cells reached about 80% confluence. Only about 10–12% of all cells appeared to be dead on the last day of culture. They were deformed and detached from the dish bottom. In the majority of cells, the nuclei were elliptical and did not have features suggestive of degeneration. On the 13^th^ day, 56 ± 0.9% of all cells were identified as microglia. Microglia were identified using a CD11b (microglia) MicroBeads rat cell sorting analysis performed in three experiments (n = 9). A one-way ANOVA revealed group differences in the percentage of microglia in the studied mixed glial cultures prepared from the brains of pups (*F*_(3,33)_ = 56.37; *p* < 0.0001). Microscopic observations performed on five mixed glial cultures gave a similar image (see **Figure 1A**). Morphological analysis of nuclei in microglial cells stained with Hoechst 33342 did not indicate deformation, apoptotic bodies, nor pyknosis (**Figure 1B**). Cells stained with agglutinin-1 in microglia culture were tightly packed. Round-shaped cells were situated in the top layer and less visible branched-ramified resting forms were also present (**Figure 1C**).

### Glial Cultures Prepared From the Offspring of Pregnant Dams Exposed to Oral Gavage Administration of Water

Prenatal stress induced morphological modifications. The cells also formed numerous aggregations. Up until the eighth day, 35–47% of cells had the characteristics of dying cells, i.e., the cells were deformed and shrunken and had a tendency to detach from the dish bottom. The live cells, in contrast, exhibited polymorphic forms. Single giant cells were observed. Starting at the eighth or ninth day, the cell culture became coherent and the dynamics of confluence were visible. On the 13^th^ day, 67 ± 1.5% of all cells were identified as microglia using a CD11b (microglia) MicroBeads cell sorting analysis performed in three experiments (n = 9). This observation suggests that the percentage of microglia in the vehicle-treated group was higher than in the control group (*p* < 0.0001, Bonferroni *post hoc* test). Microscopic observations were made on three mixed glial cultures and yielded similar images (**Figure 1D**). Staining of microglia separated from the mixed glial cultures using Hoechst 33342 confirmed features of pyknosis in about 35% of the observed cells. In some cells, deformation of nuclei and interrupted membrane integrity were noted (**Figure 1E**). Agglutinin-1-positive cells were enlarged (**Figure 1F**).

### Glial Cultures Prepared From the Offspring of Pregnant Dams Exposed to Oral Gavage Administration of IMI (10 mg/kg)

Up until day 8 or 9, the cells exhibited a tendency to detach from the dish bottom and formed numerous aggregations. Adherent cells showed a substantial morphological polymorphism that was a characteristic feature of these cultures. In particular, many cells had a longer soma with few cellular processes, thus resembling “neuron-like morphology.” There were also enlarged multinuclear cells. Up until the eighth day, about 60% of cells showed features that are characteristic of dying cells. Up until the 13^th^ day, cells covered the bottom of the dish; however, morphological polymorphisms and degeneration of the nuclei remained visible. Cells in the stress+IMI culture became coherent later than in the control cultures. On the 13^th^ day, cell division phases were rare. On day 13, 73 ± 1.6% of all cells were identified as microglia using a CD11b (microglia) MicroBeads cell sorting analysis performed in three experiments (n = 10). The percentage of microglia in the stress+IMI cultures was higher than observed in the control group (*p* < 0.0001, Bonferroni *post hoc* test), but did not differ from the prenatal stress group (*p* = 0.07). Findings of microscopic observations made on three cultures were similar (**Figure 1G**). Staining of microglia that were separated from the mixed glial cultures using Hoechst 33342 revealed the presence of pyknotic cells (about 55%) (**Figure 1H**). Polymorphic forms of agglutinin-1-positive cells were observed. These cells were characterized as enlarged cells and/or cells with a neuron-like morphology characterized by elongated soma and a few thin, branched processes (**Figure 1I**).

### Glial Cultures Prepared From the Offspring of Pregnant Dams Exposed to Oral Gavage Administration of VEN (20 mg/kg)

The dynamics of the growth culture in the stress+VEN group were similar to the control cultures. From the sixth or seventh day, the majority of cells in the stress+VEN culture were adherent. The cells did not form aggregations nor meanders. Cells covered the dish bottom and reached 90% confluence on the 13^th^ day. A greater variety of morphological forms were visible than in the control cultures. On the 13^th^ day, 50 ± 1.4% of all cells were identified as microglia using a CD11b (microglia) MicroBeads cell sorting analysis performed in three experiments (n = 9). The percentage of microglia did not differ in the stress+VEN culture as compared to the control group (*p* = 0.065, Bonferroni *post hoc* test). Findings of microscopic observations made on three cultures were similar (**Figure 1J**). According to the results of staining with Hoechst 33342, fragmented nuclei with condensed chromatin were visible only in single cells (**Figure 1K**). Cells stained with agglutinin-1 appeared similar to cells in the control cultures (**Figure 1L**).

### Viability of Microglial Cells

There were significant group differences in the percentage of living cells quantified by a cell counter Eve (*p* < 0.0001, Kruskal-Wallis test) or the MTT assay (*F*_(3,55)_ = 235.8, *p* < 0.0001, one-way ANOVA). In line with these findings, there were group differences in the amount of necrotic cells estimated using the LDH test (*F*_(3,43)_ = 230.8, *p* < 0.0001, one-way ANOVA). The use of the oral gavage during pregnancy was associated with a significant decrease in the percentage of alive cells (*p* < 0.0001) and a marked increase in the percentage of necrotic cells (*p* < 0.0001) as compared to microglia separated from the control mixed glial cultures. The application of VEN during pregnancy exerted more potent protective effects than IMI against toxicity induced by stress. In microglia exposed to stress+VEN, the percentage of alive cells estimated by the cell counter Eve (*p* < 0.001) and MTT assay (*p* < 0.0001) was higher than in the microglia in the prenatal stress group. In line with these findings, the percentage of non-alive cells was lower in the stress+VEN group as compared to the vehicle-treated group (*p* < 0.0001). In comparison to the microglia in the prenatal stress group, the percentage of alive cells was higher in the stress+IMI group as measured using the MTT assay (*p* < 0.0001). Similar results were obtained using the cell counter Eve (*p* = 0.07) and using the percentage of non-alive cells (*p* = 0.15).

There were also significant group differences in the percentage of alive cells quantified by a cell counter Eve (*p* < 0.0001, Kruskal–Wallis test) or the MTT assay (*p* < 0.0001, Kruskal–Wallis test). Similar results were obtained using the amount of necrotic cells estimated by the LDH test (*F*_(3,43)_ = 231.8, *p* < 0.0001, one-way ANOVA). Stress was shown to enhance the toxic effects of LPS (1 µg/ml) given to the culture medium for 24 h. Indeed, the percentage of alive cells was reduced (*p* < 0.0001), whereas the percentage of non-alive cells was increased (*p* < 0.0001) in comparison to LPS effects in the control microglia. The stress+VEN group was associated with a greater reduction in the toxic effects of LPS, as measured using both the cell counter Eve (*p* < 0.0001) and the LDH test (*p* < 0.001). In contrast, IMI was shown to have stronger effects than VEN on attenuating the effects of LPS. These attenuating effects were observed using the MTT assay that indicates cell viability dependent on mitochondrial activity. Indeed, percentage of viable cells was higher in microglia in the stress+IMI group as compared to the prenatal stress group exposed to LPS (*p* = 0.06) (**Table 2**).

**Table 2:** Viability of cells in microglia cultures unexposed or exposed to LPS (1μg/ml) for 24 hours.

	PRENATAL STRESS	STRESS+ IMI	STRESS+ VEN	PRENATAL STRESS	STRESS+IMI	STRESS+VEN
	medium			medium + LPS		
% of viable cells*(Cell counter Eve)*	70 ± 0,5^****^	82 ± 1,8^**^	96±0,7^+++^	71 ± 0,6^####^	79 ± 0,7^###^	90 ± 0,6^&&&&^
% of non -viable cells *(LDH assay)*	200 ± 3,9^****^	189 ± 4^****^	114 ± 2,7^ 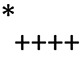 ^	203 ± 4,5^####^	179 ± 2,5^####^	114 ± 3^&&&&^
% of viable cells *(MTT assay)*	58 ± 1,3^****^	74 ± 0,8^ 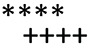 ^	89 ± 1^ 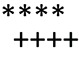 ^	48 ± 0,7^####^	55 ± 0,4	43 ± 0,3^####^

Details are as shown in **Table 1**. The results are expressed as percentage of the appropriate control or control+LPS group considered as 100 %. The percentage of alive cells was examined using the cell counter Eve or the MTT assay and the percentage of necrotic cells by the LDH test. Data are the means ± SEM of three independent experiments (n = 11 – 15). Data of cell counting were analyzed by Kruskal-Wallis test followed by Dunn's test. Results of the MTT assay were analyzed by one-way ANOVA followed by Bonferroni post hoc test or Kruskal-Wallis test followed by Dunn's test (after incubation with LPS). Data of the LDH test were analyzed by one-way ANOVA followed by Bonferroni post hoc test; * p < 0.05, ** p < 0.01,**** p < 0.0001 vs. control group; ^+++^ p < 0.001, ^++++^ p<0.0001 vs. prenatal stress group; ^###^ p < 0.001 ,^####^ p < 0.0001 vs. control+LPS group; ^&&&&^ p < 0.0001 vs. prenatal stress+LPS group.

### Synthesis and Expression of IL-1β, TNF-α, and NO in Microglia

In the supernatants of the microglia cultures, there was a significant difference in the concentration of the pro-inflammatory markers (i.e., IL-1β, TNF-α) and NO across the studied groups (IL-1β, *F*_(3,32)_ = 87.2, *p* < 0.0001, one-way ANOVA; TNF-α, *F*_(3,__32)_ = 160.4, *p* < 0.0001, one-way ANOVA; and NO, *p* < 0.0001, Kruskal–Wallis test). In particular, an increased concentration of IL-1β (*p* < 0.0001) and NO (*p* < 0.05) was found in the supernatants of cultures prepared from the brains of pups born to dams from the prenatal stress group as compared to the control group. The largest increase was observed in the secretion of NO (i.e., sevenfold increase), whereas there was only a trend for an increase in TNF-α release. Prenatal exposure to IMI (stress+IMI group) was associated with a potent increase in the release of TNF-α (*p* < 0.0001) and NO (*p* < 0.01) in comparison to the prenatal stress group. The observed enhancement in IL-1β release did not change after exposure to IMI. Exposure to VEN induced the opposite effect. In particular, following VEN, secretion of both IL-1β (*p* < 0.0001) and NO (*p* < 0.05) was reduced. Furthermore, the concentration of IL-1β tended even to be lower in the stress+VEN group as compared to the control group (*p* = 0.09). The concentration of TNF-α and production of NO were similar as in the control group.

**Figure 2:**
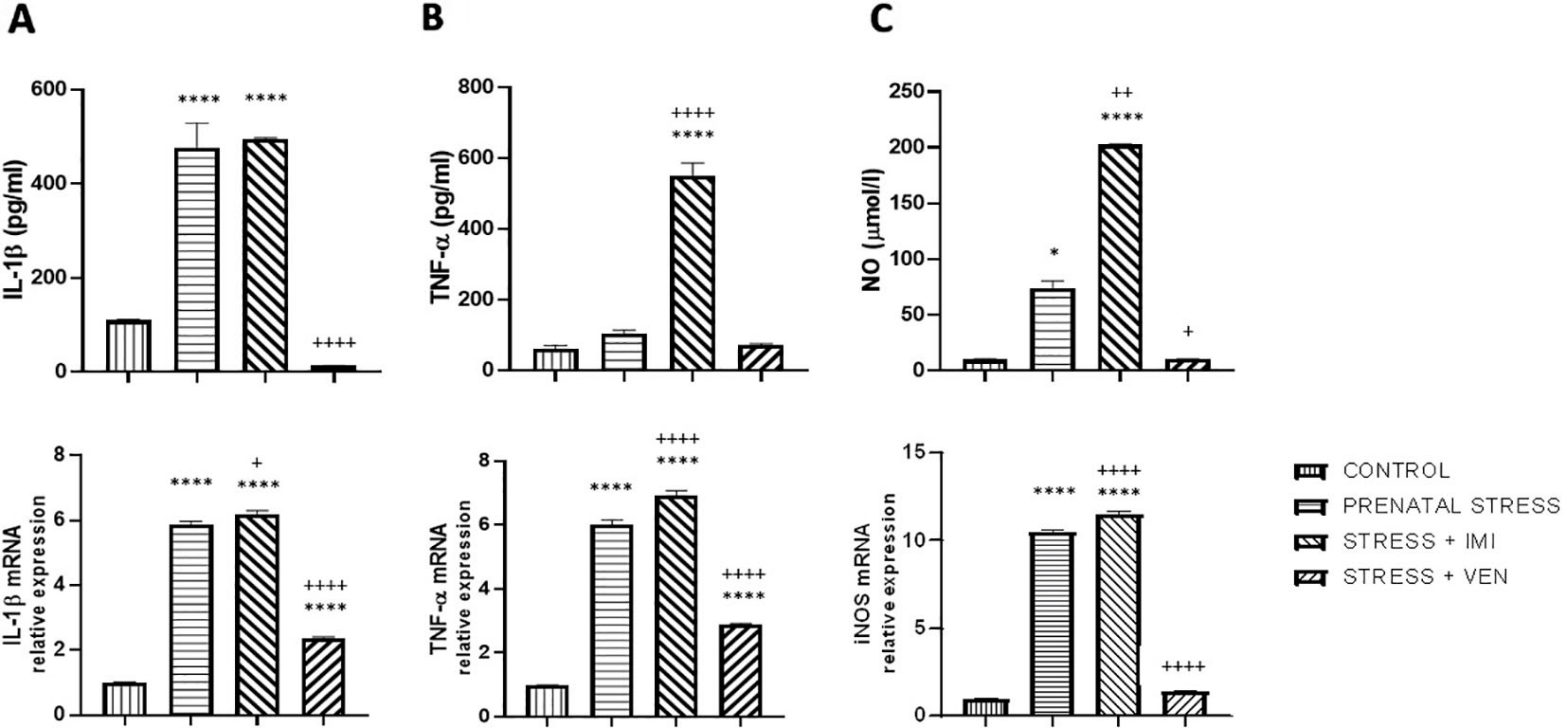
The effects of exposure to imipramine (IMI) or venlafaxine (VEN) combined with prenatal stress on IL-1β and IL-1β mRNA **(A)**, TNF-α and TNF-α mRNA **(B)**, and NO and iNOS mRNA **(C)** in primary microglial cell culture. IMI (10 mg/kg) or VEN (20 mg/kg) was administered once daily by oral gavage from gestational day (GD) 7 to GD 22. Cultures were prepared from the brains of pups delivered by dams from one of four groups: (1) drinking water during pregnancy (control group); (2) receiving water (prenatal stress group), (3) IMI (stress+IMI group), or (4) VEN (stress+VEN group) additionally once daily by oral gavage. Data are presented as mean ± SEM from three independent experiments (n = 9). Data were analyzed using a one-way ANOVA followed by Bonferroni *post hoc* test or by Kruskal–Wallis test followed by Dunn's test (NO level in culture supernatants); ^*^p < 0.05, ****p < 0.0001 vs. control group; ^+^*p* < 0.05, ^++^*p <* 0.01, ^++++^*p* < 0.0001 vs. prenatally stressed group.

There was a significant difference in mRNA expression of the tested markers (i.e., IL-1β, TNF-α, NO) across the studied groups (IL-1β, *F*_(3,32)_ = 985.3, *p* < 0.0001; TNF-α, *F*_(3,__32)_ = 761.6, *p* < 0.0001; NO, *F*_(3,32)_ = 2300, *p* < 0.0001, one-way ANOVA). In particular, there was a significant increase in expression of IL-1β mRNA (*p* < 0.0001), TNF-α mRNA (*p* < 0.0001), and iNOS mRNA (*p* < 0.0001) in the prenatal stress culture as compared to the control culture. The most potent (i.e., 10-fold) increase was observed for expression of iNOS mRNA. In comparison to the effect of prenatal stress alone, exposure to IMI only slightly enhanced the effects of stress on expression of IL-1β mRNA (*p* < 0.05). Expression of TNF-α mRNA (*p* < 0.0001) and iNOS mRNA (*p* < 0.0001), in contrast, showed a more substantial increase. In contrast, VEN evoked a suppressive effect such that exposure to VEN decreased mRNA expression for IL-1β (*p* < 0.0001), TNF-α (*p* < 0.0001), and iNOS (*p* < 0.0001) in comparison to the prenatal stress group. The expression of IL-1β mRNA (*p* < 0.0001) and TNF-α mRNA (*p* < 0.0001) was still higher than in the control cultures, whereas iNOS mRNA did not differ from levels in the control group (**Figures 2A–C**).

### Exposure of LPS to Microglia Cultures Influences the Synthesis and Expression of IL-1β, TNF-α, and NO

The effects of LPS on the secretion of pro-inflammatory cytokines (i.e., IL-1β, TNF-α) and NO differed across groups (IL-1β, *p* < 0.0001; TNF-α, *p* < 0.0001; NO, *p* < 0.0001, Kruskal–Wallis test). In particular, in the control cultures, LPS markedly increased the production of all of the studied markers as compared in the unstimulated control cultures (*p* < 0.0001, Mann–Whitney *U* test, data not shown). Prenatal stress increased the vulnerability of microglia to LPS, evidenced by an increase in concentration of the cytokines IL-1β (*p* < 0.0001), TNF-α (*p* < 0.01), and NO (*p* < 0.001) as compared to the effects of LPS in the control cultures. The observed increase in NO production was the most potent effect. Administration of IMI or VEN during pregnancy suppressed the interactive effects of stress and LPS, but to a different extent. In particular, IMI inhibited the effects of LPS on IL-1β (*p* < 0.05) and slightly decreased TNF-α production. However, IMI had no influence on the effects of LPS on NO release. Exposure to VEN evoked stronger suppressive effects. The levels of IL-1β (*p <* 0.05), TNF-α (*p* < 0.0001), and NO (*p* < 0.001) in culture supernatants were highly decreased in comparison to the effects of LPS in microglia cultures from offspring of the prenatally stressed dams. Levels of TNF-α and NO were similar to levels induced by LPS in the control culture.

**Figure 3:**
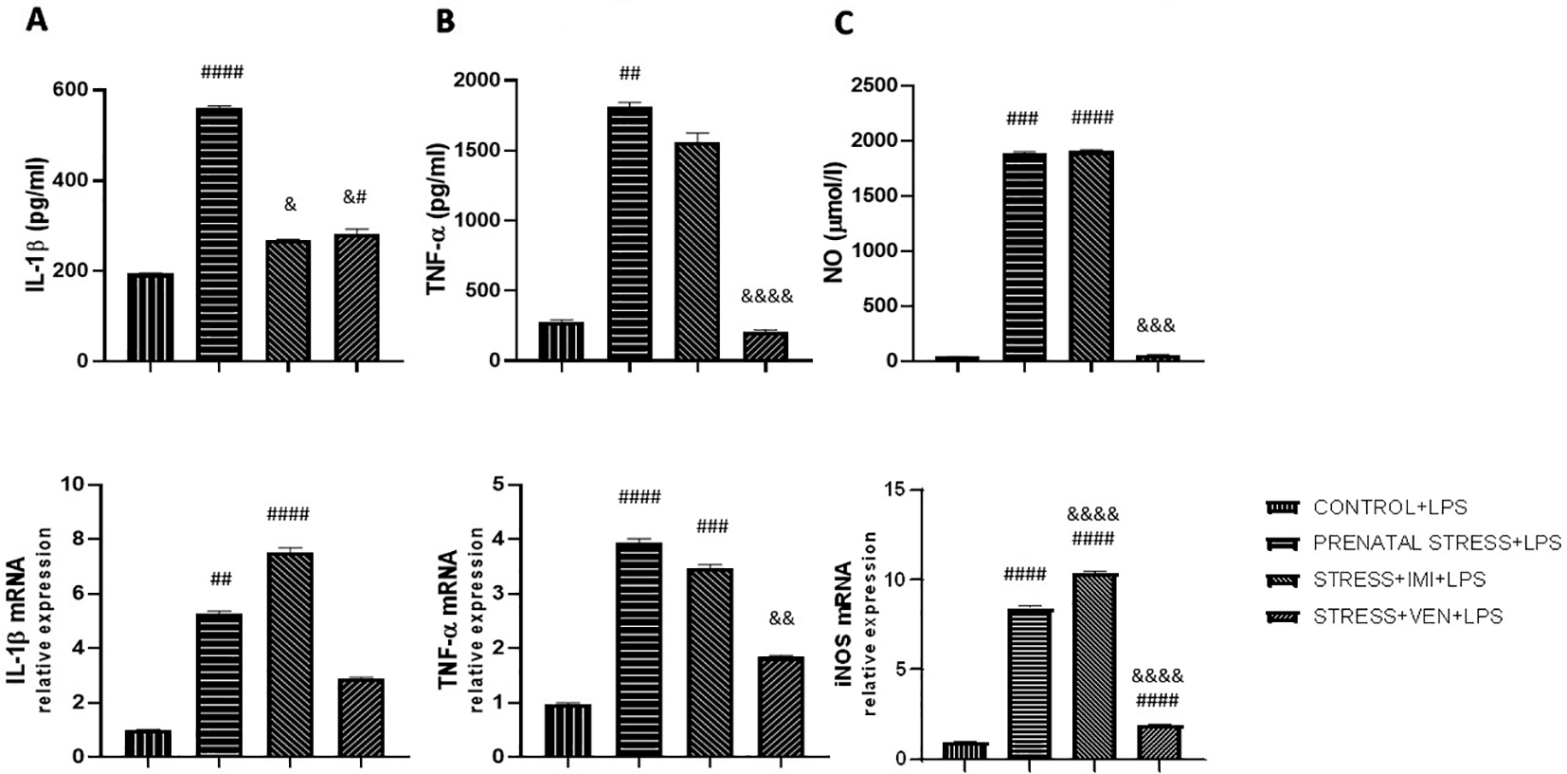
The effects of exposure to imipramine (IMI) or venlafaxine (VEN) combined with prenatal stress on LPS-stimulated IL-1β and IL-1β mRNA **(A)**, TNF-α and TNF-α mRNA **(B)**, NO and iNOS mRNA **(C)**, in primary microglial cell cultures. Experimental details are the same as in **Figure 2**. Briefly, cultures were exposed to LPS (1 µg/ml) to induce TNF-α release (for 6 h), IL-1β and NO release (for 24 h), or to stimulate TNF-α mRNA expression (for 4 h), or IL-1β or iNOS mRNA (for 12 h). Data are presented as mean ± SEM from three independent experiments (n = 9). Data were analyzed using a Kruskal–Wallis test followed by Dunn's test or with one-way ANOVA followed by a Bonferroni *post hoc* test (iNOS mRNA expression); ^#^*p <* 0.05, ^##^*p <* 0.01, ^###^*p <* 0.001, ^####^*p* < 0.0001 vs control+LPS group; ^&^*p <* 0.05, ^&&^*p <* 0.01, ^&&&^*p <* 0.001, ^&&&&^*p* < 0.0001 vs. prenatally stressed +LPS group.

Similar changes were observed in expression of the studied mRNAs. There were significant differences in the effects of LPS on the expression of IL-1β mRNA (*p* < 0.0001, Kruskal–Wallis test), TNF-α mRNA (*p* < 0.0001, Kruskal–Wallis test), and iNOS mRNA (*F*_(3,__32)_ = 2290, *p* < 0.0001, one-way ANOVA). Following LPS stimulation, mRNA expression was remarkably increased as compared to the unstimulated control cultures (*p* < 0.0001, Mann–Whitney *U* test, data not shown). The effects of LPS on mRNA of all of the studied markers were stronger in microglia derived from the offspring of prenatally stressed dams as compared to microglia in the control group (IL-1β mRNA, *p* < 0.01; TNF-α mRNA and iNOS mRNA, *p* < 0.0001). The most potent effect was the observed increase in iNOS mRNA expression. Prenatal exposure to stress and IMI induced only modest effects on the LPS-stimulated IL-1β mRNA and TNF-α mRNA expression, but was shown to enhance the effect of LPS on iNOS mRNA expression (*p* < 0.0001). Response to LPS in cultures from brains of pups subjected *in utero* to VEN was markedly reduced for expression of iNOS mRNA (*p* < 0.0001) and TNF-α mRNA (*p* < 0.01) (**Figures 3A–C**).

### Exposure of LPS to Microglia Cultures Influences Bax/Bcl2 mRNA Expression

The microglia cultures from the studied groups differed significantly in the expression of Bax/Bcl2 mRNAs ratio (*p* < 0.0001, Kruskal–Wallis test). The ratio of Bax/Bcl2 mRNA was lower in microglia cultures prepared from brains of pups exposed to prenatal stress (*p* < 0.01) as compared to the control cultures. There was a marked interaction between IMI and prenatal stress. Indeed, IMI given during pregnancy remarkably increased the ratio of Bax/Bcl2 mRNA as compared to the ratio of Bax/Bcl2 mRNA in the prenatal stress group (*p* < 0.0001). VEN induced weaker effects than IMI. In particular, there was only a trend for an increase in the ratio of Bax/Bcl2 mRNA in the VEN group as compared to the prenatal stress group. Further, the ratio of Bax/Bcl2 mRNA was lower in the stress+VEN group as compared to the control group.

**Figure 4:**
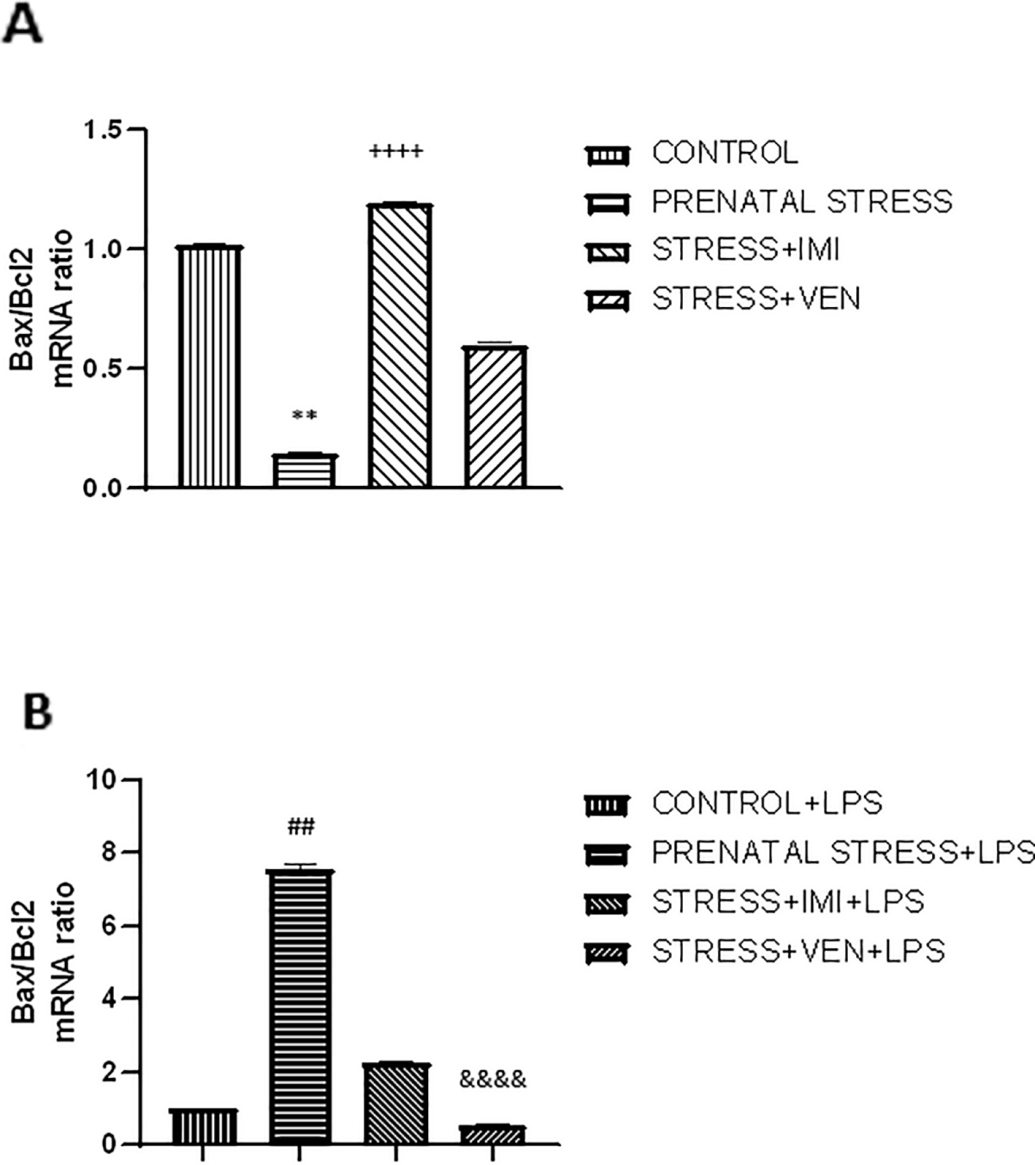
The effects of exposure to imipramine (IMI) or venlafaxine (VEN) combined with prenatal stress on the ratio of Bax/Bcl2 mRNA **(A)**, and the ratio of Bax/Bcl2 mRNA following LPS induction **(B)**. Details of the experimental procedure are shown in **Figure 2**. Microglial cultures were exposed to LPS (1 µg/ml) for 12 h. Data are presented as mean ± SEM from three independent experiments (n = 9). Results were analyzed using a Kruskal–Wallis test followed by Dunn's test; **p < 0.01 vs. control group; ^++++^*p* < 0.0001 vs. prenatally stressed group; ^##^*p* < 0.01 vs. control+LPS group; ^&&&&^*p* < 0.0001 vs. prenatally stressed +LPS group.

The expression of Bax/Bcl2 mRNA ratio following LPS *in vitro* stimulation was different across the studied groups (*p* < 0.0001, Kruskal–Wallis test). Incubation with LPS was associated with the shift in the balance between Bax and Bcl2 mRNAs in favor of Bax. LPS caused an increase in Bax/Bcl2 mRNA ratio in the control culture (*p* < 0.0001, Mann–Whitney *U* test, data not shown), and the ratio was even higher in cultures prepared from prenatally stressed pups (*p* < 0.01). Both drugs given during pregnancy modulated the response of microglial cells to LPS. In particular, IMI attenuated the effect of LPS, but VEN strongly ameliorated the Bax/Bcl2 mRNA ratio in comparison to LPS effects in the prenatal stress group (*p* < 0.0001) (**Figures 4A, B**).

### Exposure of LPS to Microglia Cultures Influences CX3CR1 mRNA Expression

The expression of CX3CR1 mRNA significantly differed across the studied groups (*p* < 0.0001, Kruskal–Wallis test). Indeed, prenatal stress markedly increased the expression of CX3CR1 mRNA in the microglial cells (*p* < 0.0001) relative to control conditions. Further, IMI blocked the effects of stress (*p* < 0.0001), such that expression of CX3CR1 mRNA was similar to what was observed in the control group. VEN attenuated the effects of stress, but CX3CR1 mRNA expression was still higher than in the control group (*p* < 0.05).

**Figure 5:**
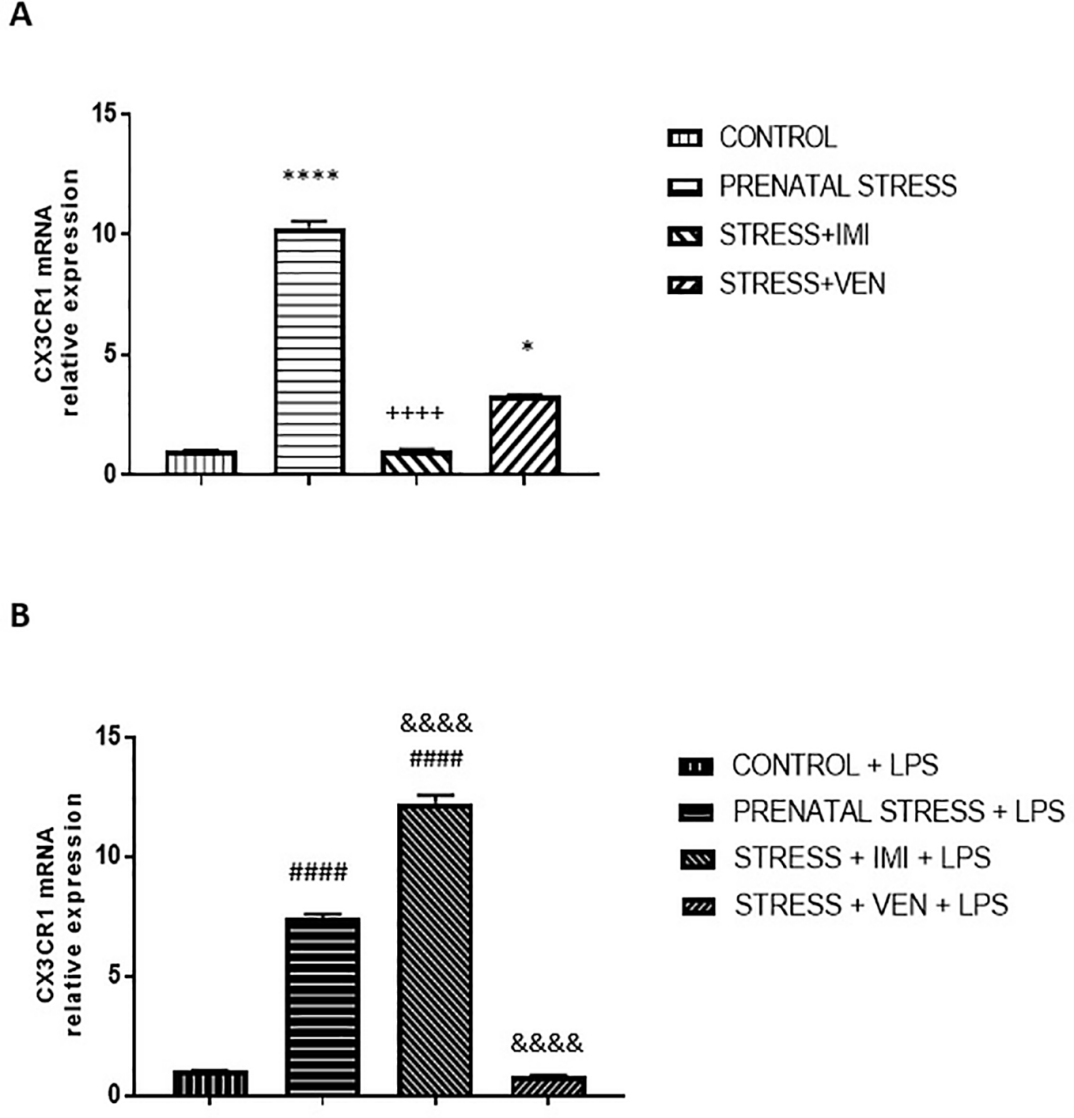
The effects of exposure to imipramine (IMI) or venlafaxine (VEN) combined with prenatal stress on the CX3CR1 mRNA **(A)**, and CX3CR1 mRNA following LPS induction **(B)**. The primary microglial cell cultures were exposed to LPS (1 µg/ml) for 12 h. Experimental details are provided in **Figure 2**. Data are presented as mean ± SEM from three independent experiments (n = 9). Data were analyzed using a Kruskal–Wallis test followed by Dunn's test. The effects of LPS on CX3CR1 mRNA expression were examined using a one way-ANOVA followed by Bonferroni *post hoc* test; * *p* < 0.05, ^****^p < 0.0001 vs. control group; ^++++^*p* < 0.0001 vs. prenatally stressed group; ^####^*p* < 0.0001 vs. control+LPS group; ^&&&&^*p* < 0.0001 vs. prenatally stressed+LPS group.

CX3CR1 mRNA expression was enhanced in all of the microglia cultures treated with LPS as compared to unstimulated cultures (*p* < 0.0001, Mann–Whitney *U* test or Student *t*-test, data not shown). Indeed, LPS altered CX3CR1 mRNA expression in all groups (*F*_(3,__32)_ = 903.3, *p* < 0.0001, one-way ANOVA). However, the extent to which CX3CR1 mRNA expression was enhanced differed across groups. *Post hoc* analysis showed that LPS induced a large increase in mRNA expression in cultures from pups of the prenatally stressed dams (*p* < 0.0001 vs. control+LPS) and a very large increase in cultures prepared from brains of pups treated with IMI *via* their dams (*p* < 0.0001 vs. the prenatal stress+LPS group and vs. the control+LPS group). In contrast, VEN administered during pregnancy markedly reduced microglial response to LPS (*p* < 0.0001; vs. the prenatal stress+LPS group). In these cultures, CX3CR1 mRNA expression did not differ from levels measured in the LPS-stimulated control microglia (**Figures 5A, B**).

## Discussion

Here, we found that oral gavage administration once daily from GD 7 to GD 22 is associated with stress. This prenatal stress did not affect pregnancy outcomes, but did have an impact on the behavior of pups. We observed a disrupted surface righting reflex in rat pups exposed to prenatal stress. Importantly, prenatal stress affected the morphology of glial cells and microglial signaling. In comparison to control cultures, mixed glia cultures prepared from pups exposed to stress were characterized by an enhanced percentage of microglial cells, an increased proportion of ameboid microglial cells and polymorphic forms, and an increased percentage of dying microglial cells. However, the expression of Bax/Bcl2 mRNAs ratio was lower in comparison to expression in the control microglia cultures. The observed marked increase in the percentage of nonviable microglial cells was confirmed by the LDH test. Microglia activation caused by chronic mild prenatal stress was characterized by increased production of IL-1β and NO. To a large extent, we found that exposure to prenatal stress increased vulnerability of microglia to LPS, as measured by all of the studied markers (i.e., IL-1β, TNF-α, NO, Bax/Bcl2 mRNA).

Microglia are considered to be the neuro-immune sensors of stress (Frank et al., 2019). However, to date, the influence of prenatal stress on microglia has only been investigated in a few studies. For the first time, Ślusarczyk et al. (2015) demonstrated overactivation of microglia in cultures obtained from the brains of rat pups exposed to stress applied as an animal model of depression. They detected an increase in the proportion of ameboid cells and an increase in the production of NO, pro-inflammatory cytokines (i.e., IL-1β, TNF-α, IL-6, IL-18), and chemokines in the microglia culture evaluated 2 days after plating. We found a similar pattern of microglia alterations induced by longer exposure to a weaker repeated stressor, namely, from GD7 to GD22 vs. GD14 to GD22. We applied the stressor before microglia invasion into the embryonic brain (Alliot et al., 1999). The abovementioned alterations we detected in microglial activity later, in cells separated on day 13 from the mixed glial cultures. The mechanism underlying the observed prolonged effects of prenatal stress on microglia has not yet been identified. Aside from changes in gene transcription, there are some data to suggest that epigenetic mechanisms involving DNA methylation, histone modifications, and noncoding RNAs play a role in modulating the inflammatory response of microglia (Garden, 2013).

Prenatal stress induced impairments in microglia signaling. Of the studied markers, IL-1β and NO showed the most marked increase. There is ample evidence to suggest that IL-1β plays an essential role in mediating the stress response (Goshen and Yirmiya, 2009). Further, evidence suggests that overproduction of IL-1β can lead to the priming of microglia. As a master regulator of neuroinflammation, IL-1β induces the production of toxic mediators such as pro-inflammatory cytokines, prostaglandins, reactive oxygen and nitrogen species, and proteases. IL-1β has been shown to not only stimulate microglia activation but also initiate the infiltration of leukocytes into the brain (Basu et al., 2004). When overproduced, IL-1β has diverse actions, including affecting neuronal function (Dunn, 2006), inducing apoptosis (Cai et al., 2004), and diminishing neurogenesis (Koo and Duman, 2008). In response to stress, an increase in the expression of iNOS mRNA and an increase in the production of NO production were detected. iNOS is an NOS isoform that is activated by an inflammatory stimulus. iNOS is primarily responsible for NO production in activated microglial cells. In the CNS, NO has a dual role; at low levels, NO supports neuronal function, synaptic transmission, neuronal survival, and energy homeostasis. At high concentrations, in contrast, NO has been shown to trigger cell-destructive processes. The overproduction of NO by activated microglia leads to the formation of peroxynitrite, which kills cells by disturbing mitochondrial function (Pacher et al., 2007).

The results of several studies indicate that the alterations in microglia evoked by prenatal stress persist in the brain and are observed in adulthood. In prepubertal or adult offspring exposed to prenatal stress, an increased number of microglia with altered morphology (i.e., an increased number of cells type III or amoeboid cells) in the hippocampus and frontal cortex have been reported (Diz-Chaves et al., 2012; Ślusarczyk et al., 2015; Zhao et al., 2015). These studies also reported increased expression of classical microglial activation markers (Diz-Chaves et al., 2012; Zhao et al., 2015) and reduced expression of alternative activation markers in the hippocampus (Zhao et al., 2015). This apparent dysregulation in microglial signaling was accompanied by deficits in spatial learning and memory, which were reversed by inhibition of microglia pro-inflammatory activation (Zhao et al., 2015).

In the present study, we observed an increased percentage of dying cells in microglia derived from brains of prenatally stressed pups. This observation was confirmed by the results of LDH test and staining with Hoechst 33342 dye that were parallel with the microscopic observations of the mixed glial cultures. Despite these observations, the ratio of Bax/Bcl2 mRNA was even lower than observed in the control culture. Both of these proteins—the proapoptotic Bax and the antiapoptotic Bcl2—have been shown to regulate mitochondrial membrane permeability during apoptosis. A shift in the balance between these proteins towards Bcl2 may reflect adaptive changes in response to the harmful effects of mild chronic stress. The impact of prenatal stress on microglia apoptosis, and its underlying mechanisms, remains unclear. Apoptotic mechanisms are highly complex (Elmore, 2007). It is plausible that another pathway, aside from the intrinsic pathway, may have been responsible for the observed apoptotic stress effect. For instance, an increased production of the NLRP3 inflammasome has been reported to mediate chronic stress effects *via* immune activation (Zhang et al., 2015). The NLRP3 inflammasome is interesting as a potential mediator of alterations induced by prenatal stress in microglial cells, because it (1) exists only in microglia (Gustin et al., 2015), (2) is involved in the cell-death process, and (3) triggers the release of IL-1β.

The results of the present *ex vivo* study are consistent with the results of prior studies (Diz-Chaves et al., 2012; Szczesny et al., 2014) showing that prenatal stress can enhance the response of the brain to LPS, as measured by an increase in the production of pro-inflammatory cytokines in adult offspring. Our data indicate that the exaggerated response to LPS is mediated by long-lasting changes induced by prenatal stress in microglia that were separated from the mixed glia cultures on day 13. The cellular mechanism responsible for this effect is unknown. Previous research indicates that the different response to LPS found in the hippocampi of prenatally stressed rats was not caused by changes in the expression of toll-like receptor 4 (TLR4), which has been shown to play a key role in LPS signaling (Diz-Chaves et al., 2012). We report, for the first time, an increased reactivity of microglia from the stressed pups to LPS. This result suggests that exposure to chronic, even mild forms of prenatal stress can sensitize microglia to immune challenges (e.g., infections). Immune challenges can occur during fetal development and consequently lead to enhanced neuronal damage in the embryonic brain (Paolicelli and Ferretti, 2017). Importantly, the prior studies by Diz-Chaves et al. (2012) and Szczesny et al. (2014) highlight that prenatally challenged microglia remained primed into adulthood. These data underscore the significance of microglia alterations induced by prenatal stress and suggest a role for microglial alterations in risk of affective disorders during adolescence or adulthood (Czéh and Nagy, 2018). Moreover, our results confirm previous reports (Liu et al., 2001) showing an overactivation of microglia by LPS, manifested by a high production of proinflammatory cytokines (i.e., IL-1β, TNF-α) and NO. These effects resulted in an enhanced proapoptotic response. In particular, we detected an increase in the expression of Bax mRNA in comparison to Bcl2 mRNA.

The present study is the first to report an interaction between prenatal stress and IMI or VEN. We found that exposure to IMI under stress conditions induced detrimental effects on glial cells. The mixed glia culture derived from pups exposed to IMI showed substantial morphological changes. The presence of microglial cells with “neuronal morphology” (i.e., cells with elongated soma and few processes) confirmed our previous observations in *in vitro* studies (Obuchowicz et al., 2014; Kedracka-Krok et al., 2018) that IMI induces the phenotypic transition of microglial cells. Taken together, our findings indicate that exposure to IMI under prenatal stress conditions induced excessive proinflammatory activation of microglia and stimulated the overproduction of NO. The percentage of microglia in the mixed culture was higher than in the other groups. In comparison to microglia from the stressed pups, the production of TNF-α and NO was remarkably enhanced, and IL-1β production remained increased in response to IMI. In contrast, an *in vitro* study conducted on a primary mixed glial culture showed that IMI given at a concentration of 10 µM did not alter the constitutive levels of IL-1β and TNF-α mRNA (Obuchowicz et al., 2014) and decreased production of NO (Hashioka et al., 2007; Hwang et al., 2008). As mentioned above, a wealth of evidence indicates that overproduction of IL-1β and NO induce toxic effects in the CNS. In addition, increased production of TNF-α contributes to neurotoxicity and stimulates microglia that, when activated, are a principal source of toxic substances and recruit immune cells into the CNS, thus enhancing neuroinflammation (Goncharova and Tarakanov, 2007). The antidepressant effects of etanercept, which blocks TNF-α, supports the hypothesis that TNF-α plays a causal role in the pathophysiology of depression (Krügel et al., 2013).

In the stress+IMI group, the percentage of dying cells was the highest among the studied mixed glia cultures. Further, staining with Hoechst 33342 confirmed an increased percentage of pyknotic cells in microglia. Similarly, the percentage of nonviable cells in microglia was high, as measured using the LDH test. IMI reversed the effects of stress on the mitochondria-dependent apoptotic pathway. In particular, we found that the ratio Bax/Bcl2 mRNA was higher than in microglia from the prenatally stressed group because of the shift in balance towards Bax mRNA expression (i.e., a threefold increase in Bax mRNA expression and a more than twofold decrease in Bcl2 mRNA expression). An increased production of TNF-α in microglia exposed to IMI as compared to microglia exposed to stress may underlie the reported apoptotic effect of IMI because TNF-α is a well-known inducer of the extrinsic apoptotic pathway (Elmore, 2007).

The observed harmful microglia alterations induced by *in utero* exposure to IMI under prenatal stress may contribute to the reported teratogenic effects of IMI. In line with this hypothesis, a lower number of neurons in the fetal frontal lobe were found after IMI administration from GD 9 to GD 11 (Swerts et al., 2010). In two behavioral tests, we did not observe a disturbance in the sensorimotor functioning of pups exposed to IMI; however, future studies should perform a more thorough evaluation of sensorimotor functioning. In this group of pups, we found diminished grip strength and reduced bodyweight that is indicative of poor physical development.

IMI reduced the response of microglia to LPS, manifested by a marked reduction in IL-1β and a slight reduction in TNF-α release. These results are similar to data reported earlier in a prior *in vitro* investigation that showed the suppressing effects of IMI on the LPS-stimulated production of the pro-inflammatory cytokines IL-1β and TNF-α (Hashioka et al., 2007; Hwang et al., 2008; Obuchowicz et al., 2014). The observed suppressing effects of IMI may partly result from an inhibition of NF-kB and p38MAPK activation induced by LPS (Hwang et al., 2008). Following IMI, there was about a threefold reduction in the ratio of Bax/Bcl2 mRNA in comparison to the ratio detected in the LPS-stimulated microglia derived from the stressed pups. This reduction was due to a reduction in expression of Bax mRNA, in particular. These data suggest that alterations in microglia induced by IMI caused a reduction in the pro-apoptotic effects, measured at the mitochondrial level. These apoptotic effects resulted from a short incubation of microglia with LPS. Results of the MTT assay (that measures activity of mitochondrial dehydrogenase) suggest an influence of IMI on the mitochondria of microglial cells under prenatal stress conditions. The findings presented by Réus et al. (2012) also support the hypothesis that IMI modulates mitochondrial function in the rat brain. It has also been shown that mitochondrial reactive oxygen species mediate the pro-inflammatory effects of LPS in microglia (Park et al., 2015). Given these results, the effects of antidepressants (not only IMI) on mitochondrial function, inflammatory processes, and apoptosis in microglia exposed to prenatal stress should be elucidated to better understand the molecular mechanisms underlying these processes.

Results of the present study indicate that VEN can induce protective effects against the impact of prenatal stress on glia cells. Indeed, we found that the dynamics of growth of the mixed glia culture prepared from brains of prenatal stressed pups exposed to VEN were similar to those observed in the control culture. Nuclear deformation was observed only in single cells. The percentage of microglia in the mixed glial cultures, the percentage of non-viable cells (determined *via* an LDH assay), and the percentage of viable cells (quantified by the cell counter Eve) in microglia were similar as compared to the corresponding control cultures. VEN suppressed the effects of stress on microglia activation. Production and release of IL-1β, TNF-α, and NO were greatly reduced in comparison to microglia from pups exposed only to stress. The ratio of Bax/Bcl2 mRNA was about two times lower than in IMI group, which was due to a more than twofold increase in Bcl2 mRNA expression. Consistent with these findings, a previous study found upregulated expression of Bcl-xL and downregulated expression of Bax in the hippocampus of rats subjected to chronic mild stress as a result of treatment with VEN (Wang et al., 2011). In turn, Singh et al. (2015) observed a dose-dependent apoptotic neurodegeneration in fetal neocortex after prenatal administration of VEN at the higher doses than in our study, which were selected to mimic the upper range of therapeutic recommended doses.

Our data indicate that VEN blocked microglia response to LPS in all of the studied markers (i.e., IL-1β, TNF-α, NO). The ratio of Bax/Bcl2 mRNA was about 14 times lower than in the LPS stimulated microglia that were derived from the stressed pups. This latter result is in line with previous findings showing that VEN can prevent the LPS-induced depolarization of the mitochondrial membrane in BV2 microglial cells, and thus can protect against the induction of apoptosis in activated microglia (Dubovický et al., 2014).

Furthermore, in the present study, we demonstrated alterations in CX3CR1 mRNA expression induced by the applied experimental model. In the CNS, CX3-chemokine ligand 1 (CX3CL1, fractalkine) forms a unique communication system between neurons and glial cells. CX3CL1 is a substance that is produced primarily by neurons and acts *via* binding to CX3CR1 receptors expressed on microglia (Harrison et al., 1998; Boddeke et al., 1999). It has been evidenced that, by regulating the functions of microglia, the CX3CL1-CX3CR1 system plays an important role in brain development (Arnoux and Audinat, 2015). To the best of our knowledge, data concerning the impact of prenatal stress on the CX3CL1-CX3CR1 system in glial cells are scarce. Further, how this system is modulated by *in utero* exposure to antidepressants has not yet been established. We found an increase in the expression of CX3CR1 in microglia separated from prenatally stressed neonates. Similar results were reported by Sowa et al. (2017), namely, an upregulation of CX3CL1 production in astroglial cell cultures prepared from the brains of neonates exposed to more intense prenatal stress applied as a model of depression. A large body of evidence indicates that the CX3CL1-CX3CR1 system serves as an “off” signal for the production of pro-inflammatory and neurotoxic factors. In microglia cultures, CX3CL1 has been shown to attenuate LPS stimulated release of pro-inflammatory cytokines such as IL-1β (Lyons et al., 2009), TNF-α (Zujovic et al., 2000; Mizuno et al., 2003), or NO production (Mizuno et al., 2003). The results of experiments with anti-fractalkine antibodies showed that tonic activation of CX3CR1 by endogenous CX3CL1 was able to silence an LPS-overactivated microglia (Zujovic et al., 2000). Furthermore, some *in vivo* experiments have demonstrated anti-inflammatory properties of CX3CL1 (Ślusarczyk et al., 2016) and pro-inflammatory effects of disruption in CX3CL1 signaling (Rogers et al., 2011). Taking these data into consideration, the observed increase in expression of CX3CR1 in microglia of prenatally stressed pups may be a sign of enhanced activity of the CX3CL1 system, which may have developed as a compensatory mechanism in response to chronic, moderate overproduction of IL-1β and NO. We observed differential effects of both antidepressants (i.e., IMI and VEN) given during pregnancy on CX3CR1 expression.

IMI under prenatal stress conditions markedly reduced CX3CR1 expression in microglia and induced strong pro-inflammatory and toxic effects. VEN, in contrast, had protective effects on microglia and attenuated CX3CR1 expression. However, CX3CR1 levels were still higher than those observed in the control culture. Our results suggest an essential relationship between alterations in microglia activation and CX3CR1 expression induced by the studied antidepressants. In the light of all of the available data, our working hypothesis is that the impact of antidepressants on the CX3CL1 system might be involved in their mechanism of control of stress-induced microglia activation. Indeed, low expression of CX3CR1 seems to be accompanied by augmented microglia activation. However, Hellwig et al. (2016) reported that CX3CR1-deficient adult mice subjected to chronic despair stress were characterized by lack of microglia changes to a hyper-ramified state. Further, CX3CR1-deficient adult mice exhibited greater resistance to stress-evoked depressive-like behavior and chronic treatment with VEN as compared to wild-type mice (Hellwig et al., 2016). In fact, results of various studies indicate that the protective or toxic functions of the CX3CL1-CX3CR1 system depend on the stimuli leading to microglia activation (Arnoux and Audinat, 2015).

LPS administered to the control culture medium for 12 h caused intense microglia stimulation characterized by, among others, an upregulation of CX3CR1 expression. Stress increased the susceptibility of CX3CR1 expression to LPS. These effects were concomitant with the effects observed for all of the studied pro-inflammatory and toxic factors. Microglia derived from the IMI group were characterized by the lowest levels of CX3CR1 expression prior to incubation with LPS. In these cells, LPS enhanced production of NO and TNF-α. These findings are in agreement with previous findings showing the opposite effect in microglia wherein CX3CL1 inhibits TNF-α and NO production in response to LPS (Zujovic et al., 2000; Mizuno et al., 2003). In turn, Lyons et al. (2009) reported dose-dependent effects of CX3CL1 on the suppression of IL-1β production. In microglia exposed to IMI and treated with LPS for 24 h, we noted a reduction of IL-1β release. These data may suggest that the sensitivity and duration of the influence of the increased activity in the CX3CL1 system has significance. Previously, Corona et al. (2010) found an increased and prolonged IL-1β production in CX3CR1-deficient mice after a peripheral challenge with LPS. Molteni et al. (2013) reported that agomelatine given chronically by oral gavage reduced the upregulation of pro-inflammatory cytokines (i.e., IL-1β, IL-6) in the hippocampus of adult rats and prevented the decrease in CX3CL1 and CX3CR1 production induced by acute LPS administration. In our study, in contrast, exposure to VEN reduced all of the observed LPS effects, but to a different extent.

In summary, this study showed for the first time that treatment with IMI and VEN combined with prenatal stress from GD 7 (before microglia invasion into the embryonic brain) to the last day of pregnancy elicited strong and differential effects on the morphology of the mixed glia culture and activity of microglial cells. Under prenatal mild chronic stress conditions, IMI evoked toxic effects. These toxic effects were characterized by an increase in the production of the studied pro-inflammatory, apoptotic, and neurotoxic factors, and a decrease in the expression of CX3CR1 mRNA. Of note, with respect to the effects on mRNA expression of pro-inflammatory cytokines, these findings are the opposite of the results of *in vitro* studies (Obuchowicz et al., 2014). In particular, exposure to VEN induced a marked protective effect on microglia that was even stronger than its effects reported using an astroglia–microglia co-culture with a different percentage of microglia (Vollmar et al., 2008). Except for IL-1β release, the response to LPS by microglia exposed prenatally to IMI and stress was not diminished, which is opposite to effects of IMI alone determined in *in vitro* studies. The reason for this discrepancy is likely multifactorial, but undoubtedly the interaction between IMI and prenatal stress on LPS effects was significant. Prenatal exposure to VEN had a stronger ameliorating effect on LPS-induced alterations in microglia than has been found in *in vitro* studies (see *Introduction*).

A growing number of studies indicate that microglia are essential regulators of CNS development. Microglia are involved in several important processes, such as control of neuronal death, survival of developing neurons, proliferation of neuronal progenitor cells, activity-dependent synaptic pruning, control of synapse function, and maturation. Microglia have been also proposed to have an impact on angiogenesis, astrogenesis, and oligodendrogenesis during the prenatal and early postnatal period of life (Reemst et al., 2016). In turn, the CX3CL1-CX3CR1 system determines the development of the CNS by controlling microglia function and their recruitment at the specific regions of the developing structures (Arnoux and Audinat, 2015). Our data suggest that exposure to even mild chronic prenatal stress has adverse effects on the phenotype of microglia. The observed effects on microglia may be a predisposing factor for psychiatric and neurodevelopmental disorders (Mosser et al., 2017; Tay et al., 2018). Moreover, the observed detrimental impact of IMI on the phenotype of microglia under prenatal stress conditions may help to explain, in part, the potential teratogenic effects of IMI reported in the literature. Based on these data it can be hypothesized that the harmful effects of IMI on the neurodevelopment of babies may occur especially when this drug is taken by mothers exposed to stress and trauma during pregnancy. In opposite, VEN as a drug able to reverse changes in glial cultures induced by prenatal stress may be expected to elicit the beneficial effects.

## Data Availability Statement

The datasets generated for this study are available on request to the corresponding author.

## Ethics Statement

The animal study was reviewed and approved by the Local Ethics Committee for the Care and Use of Laboratory Animals (Katowice, Poland).

## Author Contributions

EO was responsible for the conception and design of the study and wrote the manuscript. AB-W prepared cell cultures, conducted all experiments on cultures, and helped to write the manuscript. MZ, AB-W, GM, and TL carried out biochemical analyses of the samples. MG conducted behavioral tests, performed statistical analysis of all results, and prepared figures. All authors gave approval of the final version of manuscript.

## Funding

This work was supported by funds (KNW-2-021/N/5/N and KNW-2-017/N/6/N) from the Medical University of Silesia, Katowice, Poland. Publication charge was supported by the Medical University of Silesia in Katowice, Poland.

## Conflict of Interest

The authors declare that the research was conducted in the absence of any commercial or financial relationships that could be construed as a potential conflict of interest.
